# Supplementary figures and images for: Optimization of high-channel count, switch matrices for multinuclear, high-field MRI
Source: PLoS One. 2020 Aug 17;15(8):e0237494. doi: 10.1371/journal.pone.0237494 (PMC7430713; doi:10.1371/journal.pone.0237494)

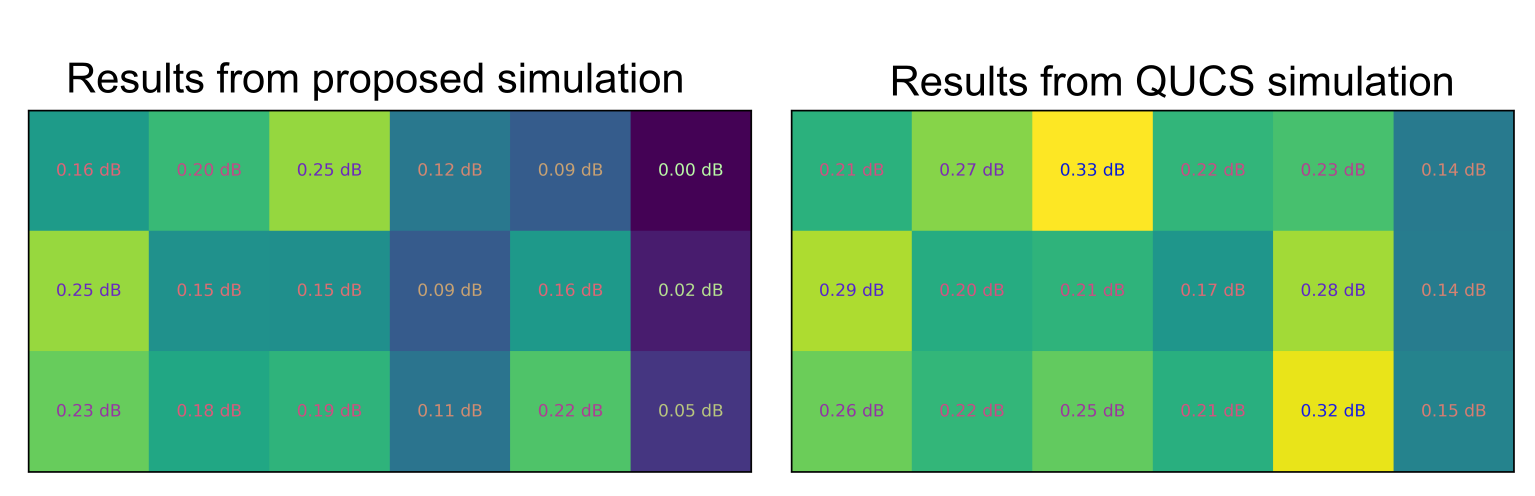

Supplement: S4 Fig — Both plots are normalized with respect to their maximum IL in color coding. While the pattern looks similar the circuit simulator predicts slightly larger insertion losses in all cases, which is probably due to using slightly different formulae for calculating transmission line properties. (PNG) [file pone.0237494.s004.png]
