# Supplementary material for: Optimization of high-channel count, switch matrices for multinuclear, high-field MRI
Source: PLoS One. 2020 Aug 17;15(8):e0237494. doi: 10.1371/journal.pone.0237494 (PMC7430713; doi:10.1371/journal.pone.0237494)
Supplement: Attachment — Submitted filename: review_P_2020.pdf [file pone.0237494.s005.pdf]

The authors present a novel design methodology for a broadband switch matrix. Its frequency is ranging from 54 to 400 MHz. As it is seen in fig 1, it seems that its hardware does not require any novelty to make the design in broadband as the authors have published the design of the PIN-diode based switch matrix before (ref 19). The novelty in PIN-diode based switch matrix design is not clear in this manuscript. As the authors mentioned mostly in the introduction section on hardware design parameters, this manuscript is on various optimization algorithms employed to optimize the performance of the switch matric design. The choice of performance parameters demonstrated in this manuscript was not clearly explained in the methods section and its motivation was not clearly given in the introduction and discussion sections. The combination of design and optimization methods in the switch matrix was shown in the manuscript but its translation into the MRI image quality and image acquisition speed is missing in the manuscript.
In general, the used language in this manuscript is adequate but it contains a few grammatical errors. The relevant referencing was sometimes missing in the introduction. Unfortunately, the image quality of the figures in the pdf is very bad, so I have to ask to the authors to improve the quality of all figures in the manuscript. The tables require further explanations in the caption since the parameters and abbreviations were not included. My detailed comments and questions are below:

1) Line 39: could the authors define “patient compliance”?
2) Line 39-40: could the authors explain further what they meant with “.. optimizing the use of expensive MRI machinery”? Is it related elaborate MRI hardware cost?
3) Line 40: Please correct double “not”.
4) Line 42: Please clarify the type of artifacts for the sentence “… correcting unwanted artifacts”
5) Line44: Could the authors define the “spectrometer”? Is it used in meaning different than the “MRI machinery”? Please explain it.
6) Line 46,47: The sentence starting with “ Thus, quite frequently..” is not clear in the context. To make it more clear, please define “receive elements” and “receiver channels”. Please provide related references.
7) Line 51: Please define the “receive power”. It is not clear here its meaning in the context.
8) Line 53: could the authors clarify the definition of “high channel-count coils to systems”? How is it different than “receiver elements”?
9) Line 59: The sentence “…which need to be flexibility connected to…” not clear in meaning and contains grammatical errors. Please correct it.
10) Line 61: It might be better to clarify the relation between the “coil connectors” and “receive elements”. Since “coil connectors” was being introduced as a new concept here, it would be more clear in the context.
11) Line 62: Could the authors explain what the “switch matrix” is? Please provide related references. Is it a common module in each MRI system?
12) Line 63: Please provide references for “cross-point switches”.
13) Line 65: Could the authors explain what “cross-point switch matrix implementation” is? Where are the switches physically located in the MRI system?
14) Line 67: Please provide references for the sentence “while the former implementation seems to be technologically more robust,..”.

15) Line 68: Could the authors clarify the meaning of ".. remaining open-ended transmission lines.."? Here, the what did the authors mean with "remaining" and "open-ended transmission lines" in this context?
16) Lines 72: Please explain further the benefit/importance of "compensating open transmission lines"?
17) Line 75: Please provide related references for the sentence " To date, switch matrix design has mostly been concerned with routing the signals of the proton Larmor frequency". Just for curiosity, how many proton switch matrix design implemented on MRI systems?
18) Line 75: What could be the reason(s) of the limited switch matrix design implementation on only proton? Could it be applied in broadband in frequency?
19) Line 78: Could the authors correct the sentence " As multichannel X-nucleus coil arrays provide the same array SNR benefits…"? Its meaning is not clear.
20) Line 81: Please correct the placement of "suitable" in the sentence. The meaning of the sentence is not clear.
21) Line 83: The limitations to design a switch matrix in broadband is not clear so far. Please include in the introduction section with related references.
22) Line 91: Please provide the elements of the switch matrix topology. Its detailed description is required if any changes was applied on the hardware design.
23) Fig1, Page 25: Is there any added design parameters to make the PIN-diode based switch matrix in broadband?
24) Line 149: Could the authors give the optimum "insertion loss"? What was the ideal insertion loss aimed?
25) Line 157: What is the motivation of choosing a switch matric with 128x64 channels? How is this number translated into the "receiver channels" or "receive elements"?
26) Line 237, Discussion section: this section misses the discussion of the methods used in this manuscript in broader MRI context. I strongly recommend the authors to include the contribution of this novelty into the MRI image quality and acquisition time. Even the optimization methods have been explored here, its contribution into the receiver power and receiver elements design were not addressed.
