# Supplementary material for: Optimization of high-channel count, switch matrices for multinuclear, high-field MRI
Source: PLoS One. 2020 Aug 17;15(8):e0237494. doi: 10.1371/journal.pone.0237494 (PMC7430713; doi:10.1371/journal.pone.0237494)
Supplement: Attachment — Submitted filename: Respose_To_Reviewers.docx [file pone.0237494.s006.docx]

# Additional Editor Comments:

Your manuscript was reviewed by three experts in MRI system and coil design. All the reviewers identified that the study needs to be substantially improved in order to meet publication standards. Specifically, R1 requested you to incorporate more details of the method in the current manuscript. R2 suggested you demonstrate how does proposed switch matrices translate to MR image quality, and to extend the methods section to explain design parameters clearly. R3 suggested you improve the introduction by stating the motivation behind the work and identifying the problems of existing switches explicitly. As highlighted by the reviewers, I suggest you validate the simulations by providing an experimental proof-of-concept study as referencing to prior work (Ref. 19-20) would not be sufficient. In addition, the image quality of all figures needs to be improved. In the view of reviews and my assessment of the manuscript, I recommended a major revision to give you enough time to prepare the revised manuscript improved by a proof-of-concept study.

The authors thank the reviewers and editor for their evaluation. We have carefully addressed all of the issues raised and detailed responses can be found below.

As suggested by **(R1)**, we have included more details relating to multi-objective optimization using evolutionary algorithms. As the topic of multi-objective optimization is very broad, we have added the most relevant information to the manuscript and have referred the reader to literature for a more in-depth discussion of the subject. In order to provide a better understanding of losses in the switch matrix and their impact on the image SNR, we have performed measurements using a 9.4T animal MR scanner **(R2)**. The corresponding results are displayed in a novel figure in the revised manuscript. In addition to these alterations, the motivation for the work has been established more clearly in the revised manuscript **(R3)**. This includes an extensive reworking of the “Abstract” and “Introduction” as well as the inclusion of additional references to describe the current state-of-the-art. The commercial implementation of the 9.4 T Magnetom and 7 T TERRA (both: Siemens Healthineers, Erlangen) is now mentioned in terms of the removal of the switch matrix and the hard routing of receive signals to the receivers.

We have also compared a small version (6 inputs and 3 outputs) of the optimized multinuclear switch matrix with simulation results from a circuit simulator (QUCS available from qucs.sourceforge.net) in order to validate the method. Although the results are in agreement, our calculations slightly underestimated the insertion loss. This might be caused by the different analytical formula used to calculate the transmission line formulas in our calculations and the circuit simulator.

As suggested, the source code of the python script used in this manuscript has been made publically available.

# Review Comments to the Author

## Reviewer #1:

This research paper provides a method of directly evaluating the loss in switch matrices used in receive arrays for different nuclei at 9.4T and compares different algorithms for finding terminations that minimize the insertion loss in the switching. The authors have very clearly outlined their process and detailed the results, while showing that significant improvements are obtained by the method. There is not enough details provided, and I am not specifically well informed enough, to determine if there are any issues with the implementation with the algorithms or if there was an expected result for the comparison of the different algorithms based on previous studies.

Thank you for your careful evaluation of the manuscript. As suggested, we have added additional details relating to the methods employed. These include more detailed information on how the terminating impedances are transformed along the transmission lines **(R1-1a)** and the computation of the transmission line parameters themselves **(R1-1b)**.

While we agree with your suggestion relating to the inclusion of more detail, we feel that an in-depth discussion of evolution algorithms goes beyond the scope of this manuscript. Instead, we have included a reference to a standard textbook as well as a list of active implementations of evolution algorithms in python **(R1-1c)**. Finally, in the discussion, we have added a hypothesis on why the ε-MOEA might outperform the other algorithms of the platypus code collection tested in the presented manuscript and have backed this up with a literature reference **(R1-1d)**.

There are a number of specific comments on details and changes that can be made to improve the paper:

Page 3, line 55 – change “speed up” to acceleration factor

Thank you for suggesting this formulation. We fully agree that it is more to the point. **(R1-2)**

Page 4, line 80 – this phrasing makes it seem like multinuclear arrays are a new development, and the quotes reference is not a very early or specifically monumental reference of a multinuclear array. Please find more relevant and primary references and describe the development of X-nuclei array more fully.

We agree that the formulations can easily be misinterpreted. In response, we have added references for multinuclear coils at clinical MRI frequencies **(R1-3a)**. The sentence itself has been modified to reflect that X-nucleus coils are not a new development and have, in fact, been a research topic for a considerable amount of time. We have also included a review article on the development of double-tuned ^23^Na/^1^H coil arrays for a number of field strengths and one of the latest applications in this field for an ^19^F/^1^H array. **(R1-3b)**

Page 5, line 96 – replace “Note also that, both” with “The”

The sentence has been modified as suggested. **(R1-4)**

Figure 1- it may be the output settings for this pdf, but the figures are almost illegible, thus for publication will be necessary to make sure they are all of high quality

We have modified this figure for improved clarity and image quality. We have also added a more detailed schematic of the crossbar switch to simplify its general topology for the reader.

Figure 2- this graph/figure does well to highlight the importance of modifying the resulting stubs to avoid insertion losses that would degrade noise factor However it also seems to provide evidence that a more complicated approach is not needed, as the maximum loss of 2.21dB would have very little effect on the final noise figure considering the gain of the preamplifier stage. The author’s previous publication “Signal Loss Compensation of RF Crossbar Switch Matrix System in Ultra-High Field MRI” also seems to point to this conclusion. This particular point should be addressed more explicitly. Some rough estimate of at what point the losses may result in an SNR loss of perhaps >5% and 2% for a single channel would be valuable.

The maximum loss on the (not optimized) prototype of 2.21 dB presented in Figure 2 is the maximum loss encountered in a small matrix and for the five different routings measured. The maximum IL in a larger matrix would be significantly higher and the issue of selecting “optimum” compensation elements – which, in theory, can be selected in a “trial-and-error” method – would become harder. This is mainly due to having to find suitable terminations for all switch configurations as well as all frequencies, and, in essence, is a combinatorial problem. We think that having some kind of “tool” to help in the selection – which is what we provide in the manuscript – would be useful for this tedious task.

In order to investigate the effect of insertion loss on image SNR, we have carried out a further experiment in which a variable attenuator was inserted at the position of the proposed switch matrix (behind the coil’s preamplifier). The results are displayed in the new Figure 3 **(R1-5)**. It is also possible to calculate the tolerable insertion loss using common performance parameters of RF frontend components in an MRI machine. We kindly ask the reviewer to look at the reply to reviewer #2, comment 24, below. The calculations allow for slightly higher losses in the switch matrix without significant impact on the noise figure of the receiver chain as they imply a preamplifier directly on the coil. This was not the case for the measurements presented in the revised manuscript, as the preamplifier was not integrated into the birdcage coil employed for the signal acquisition. However, the experimental setup chosen allowed the variable attenuator to be inserted without modifying the preamplifier power supply. Additionally, the birdcage coil provides a homogenous receive sensitivity for the sample and simplifies the task of selecting the region-of-interest for the calculation of the mean signal intensity.

Page 8, line 175- add comma: “This time,”

We thank the reviewer for the careful proofreading of the manuscript and have added the comma as suggested. **(R1-6)**

Figures 3-5 do not provide much interesting information as there is no way to know from the text or discussion of the algorithm why specific switch combinations had lower insertion loss compared to others. However, there’s some value in seeing the difference with all the different nuclei at once. I suggest that this figure may only need to be shown for one algorithm, and then it can be detailed what specific differences the different algorithms had. Right now, it is not detailed why any specific differences are observed. For this provided manuscript these figures 3-5 are also illegible.

We agree that the important information relating to algorithm performance is summarized in Tables 1 and 2. For this reason, as well as for improved image quality, we have opted to move the original Figures 3-5 into the Supporting Information section of the manuscript. We have validated that the figure quality is sufficient for a 4K display, on which the images are of far better quality than in the PDF version of the manuscript. **(R1-7)**

Page 12, line247- It would greatly enhance the work if some detailed explanation and discussion of why the e-MOEA algorithm performed best and where and why the other algorithms found non-optimal solutions. As this is the best performing algorithm it would also be best to detail it more explicitly in the methods.

We have added a textbook reference describing evolution algorithms as well as a reference listing packages available in python that implement these algorithms **(R1-8a)**. In the discussion section, we also hypothesised why the ε-MOEA algorithm outperforms the other algorithms of the platypus package by referencing prior work **(R1-8b)**. Since the general theory of multi-objective optimization and algorithms implemented to address these problems is very broad, we feel that a more in-depth comparison of the algorithms evaluated within the manuscript is out of the scope of the work.

Page 12, line 256- I would agree that for the particular problem investigated here the theoretically derived insertion losses for transmission lines should match very closely with experimental.

Thank you for backing up our assessment. However, as requested by the other reviewers we have also compared the insertion losses calculated in the manuscript with losses computed with circuit simulations. We have found a small underestimation of the losses in our calculations, which are probably due to the use of different formulas to calculate transmission line parameters in the circuit simulator. The comparison is shown in S4 Figure.

Page 12, Line 264- The 1250 mm x 660 mm size is very large. A reference or details on what is currently used in commercial systems should be included. Also, in should be discussed: how necessary is it that every channel is able to be mapped or switched between every output. I believe this specific set-up is actually not very applicable for this reason.

We agree that the final board size is rather large. This is why we have investigated alternative implementations using stacked switch configurations. An initial report of these was published as a conference presentation at last year’s ISMRM (Ko Y, Choi C-H, Shah NJ, Felder J. The miniaturisation and simplification of a crossbar switch matrix system using stacked switch blocks. Proc of the 27th annual meeting of the ISMRM; 2019 May, 11th-16th; Montreal, Canada.). In order to further emphasize this point, we have cited some additional references in the discussion paragraph that point out that the size of the single matrix is excessively large **(R1-10)**

Discussion points: why the different algorithms had different execution times and some idea of how much this could change with optimization.

The execution times listed in Table 2 depend on the algorithm implementation and how many computations are carried out in each iteration (or generation if the evolutional model was used). We found a good reference to estimate the computational burden of an evolutionary algorithm to be “Coello Coello CA, Van Veldhuizen DA, Lamont GB. Evolutionary algorithms for solving multi-objective problems. New York: Kluwer Academic; 2002.” which describes the algorithms in pseudo-code.

Some description in the methods, if possible, of the specific classes the algorithms fall under.

All algorithms employed are evolution strategies (ES), a variant of evolutionary computing, capable of handling multi-objective optimization problems. In the interests of clarity, we have added this definition to the revised manuscript **(R1-12a)**.

As far as more detailed information on the different algorithms is required, we feel that an in-depth discussion is beyond the scope of the current work. Wherever possible we have, therefore, added references to textbooks on the subject as well as indicated that not only the individual optimization strategy of each algorithm influences its suitability for a given optimization problem, but, to a great extent, performance is also influenced by the selection process of the best performing individuals of a population **(R1-12b)**.

More time needs to be spent formatting the references correctly and more consistently.

We apologize that the references are not formatted as specified. We had used EndNote to manage citations and hoped that it would deliver suitable formatting. Obviously, this what not the case and this has now been remedied.

## Reviewer #2

The authors present a novel design methodology for a broadband switch matrix. Its frequency is ranging from 54 to 400 MHz. As it is seen in fig 1, it seems that its hardware does not require any novelty to make the design in broadband as the authors have published the design of the PIN-diode based switch matrix before (ref 19). The novelty in PIN-diode based switch matrix design is not clear in this manuscript. As the authors mentioned mostly in the introduction section on hardware design parameters, this manuscript is on various optimization algorithms employed to optimize the performance of the switch matric design. The choice of performance parameters demonstrated in this manuscript was not clearly explained in the methods section and its motivation was not clearly given in the introduction and discussion sections. The combination of design and optimization methods in the switch matrix was shown in the manuscript but its translation into the MRI image quality and image acquisition speed is missing in the manuscript.

In general, the used language in this manuscript is adequate but it contains a few grammatical errors. The relevant referencing was sometimes missing in the introduction. Unfortunately, the image quality of the figures in the pdf is very bad, so I have to ask to the authors to improve the quality of all figures in the manuscript. The tables require further explanations in the caption since the parameters and abbreviations were not included.

Thank you for your evaluation of the manuscript. We apologize for the poor image quality in the PDF and have uploaded high-resolution images with the revised manuscript. A native English speaker has rechecked the revision. Finally, we have detailed the abbreviations in the table captions.

My detailed comments and questions are below:

1) Line 39: could the authors define “patient compliance”?

In medical imaging, the term “patient compliance” most often refers to the patient’s ability to remain immobile during image acquisition so as to avoid image deteriorations. This definition is also given by, e.g. Wikipedia:

“*In medicine, patient compliance (also adherence, capacitance) describes the degree to which a patient correctly follows medical advice. Most commonly, it refers to medication or drug compliance, but it can also apply to other situations such as medical device use […].*”

The authors have added scan duration as the reason for improved compliance in the revised manuscript. **(R2-1)**

2) Line 39-40: could the authors explain further what they meant with “.. optimizing

the use of expensive MRI machinery”? Is it related elaborate MRI hardware cost?

We apologize for this imprecise formulation. We had intended to hint at a higher patient throughput due to reduced scan time and thus a higher return on investment for the expensive MRI equipment. This has been rephrased in the revised manuscript. **(R2-2)**

3) Line 40: Please correct double “not”.

Thank you for the careful proofreading. We have deleted the double occurrence. **(R2-3)**

4) Line 42: Please clarify the type of artifacts for the sentence “... correcting unwanted

artifacts”

We have added that the most common artifacts in parallel imaging are aliasing and ghosting arising from subsampled k-space. (R2-4)

5) Line44: Could the authors define the “spectrometer”? Is it used in meaning different

than the “MRI machinery”? Please explain it.

The terms “MRI system”, “MRI machine”, “console” and “spectrometer” are used frequently and interchangeably in MRI terminology. We agree that this is confusing and might be “lab” jargon. Thus, the terms “MRI system” or “MRI machine” have been used in the revised manuscript. **(R2-5)**

6) Line 46,47: The sentence starting with “ Thus, quite frequently..” is not clear in the

context. To make it more clear, please define “receive elements” and “receiver

channels”. Please provide related references.

We have modified the sentence as suggested and have added a reference that describes the issues relating to the number of receive coils and receivers in the MRI system. **(R2-6)**

7) Line 51: Please define the “receive power”. It is not clear here its meaning in the

context.

We have rephrased this as “receive signal power” and have added an example describing receive signal combination in a quadrature array that combines the signal from two coil elements into a single receive signal – which is a similar operating principle to the referenced mode matrix. **(R2-7)**

8) Line 53: could the authors clarify the definition of “high channel-count coils to

systems”? How is it different than “receiver elements”?

We apologize for the confusing “to” in the sentence. This was an oversight and had not been removed during final editing of the initial manuscript. It has now been altered. **(R2-8)**

9) Line 59: The sentence “...which need to be flexibility connected to...” not clear in

meaning and contains grammatical errors. Please correct it.

Thank you for bringing this to our attention. We have added a rationale as to why the flexible connection of a high number of receive coil elements is often required in clinical practice. A native English speaker has also checked the sentence for grammatical correctness. **(R2-9)**

10) Line 61: It might be better to clarify the relation between the “coil connectors” and

“receive elements”. Since “coil connectors” was being introduced as a new concept

here, it would be more clear in the context.

The two terms and definitions have been further elaborated so as to clarify their meaning. In addition, the physical location of the different units in standard clinical MRI systems has been detailed so as to provide the reader with a more detailed view of the problem to be solved by the multinuclear switch matrix presented in this work. **(R2-10)**

11) Line 62: Could the authors explain what the “switch matrix” is? Please provide

related references. Is it a common module in each MRI system?

We have now included references pertaining to the definition of switch matrices (or matrix switches or crossbar matrix switches) arising from telecommunication systems. **(R2-11)**

12) Line 63: Please provide references for “cross-point switches”.

The term “cross-point switch” stems from the reference “Understanding Tree and Crosspoint Matrix Architectures,” which is referenced at the end of the corresponding sentence. For the sake of consistency, we have changed the term to "crossbar switch" during manuscript revision. This is the more general term for a switch matrix with M inputs and N outputs and is also used in telecommunications and circuit theory. **(R2-12)**

13) Line 65: Could the authors explain what “cross-point switch matrix implementation”

is? Where are the switches physically located in the MRI system?

Please refer to the above comment for the consistent naming convention. We have also included the common location of the switch matrix in MRI systems – both physically close to the magnet as well as behind the low noise preamplifier integrated into the MRI receive coil itself in the signal routing. **(R2-13)**

14) Line 67: Please provide references for the sentence “while the former

implementation seems to be technologically more robust,..”.

The reason for this statement is given in the following subordinate clause. Using integrated circuit switches in an SPDT configuration disconnects the open-ended stub lines. The use of a single PIN diode as a switch cannot archive this, as the PIN diode can connect to the vertical line but cannot break the connection to the remaining length of the vertical transmission line. We have rephrased the sentence in combination with your comment #15 for improved clarity. **(R2-14)**

15) Line 68: Could the authors clarify the meaning of “.. remaining open-ended transmission lines..”? Here, the what did the authors mean with “remaining” and “open-ended transmission lines” in this context?

We aimed to be brief in this part of the discussion in the original manuscript and apologize if this was to the detriment of clarity. In a SPST switch configuration with a single PIN diode, a connection can be made but not broken. In other words, the input transmission line can be connected to the selected output transmission line but it cannot be disconnected from the “remaining” section of the input transmission line, which runs the full physical length of the switch matrix board in order to be able to connect to all possible output lines. We hope that the rephrasing makes this clearer. **(R2-15)**

16) Lines 72: Please explain further the benefit/importance of “compensating open transmission lines”?

Open transmission line stubs are transformed to a parallel impedance at the junction of the closed crossbar switch. This is the major mechanism for signal attenuation across the switch as the impedance may be approaching a short circuit if the stub length is close to λ/4, 3λ/4 and so forth. This is why references “Lee HL. Reconfigurable multi-channel RF crossbar switch matrix for MRI receiver front-end. Electronics Letters. 2017;53(6):380-1.” and “Ko YK, Choi CH, Shah J, Felder J. Signal Loss Compensation of RF Crossbar Switch Matrix System in Ultra-High Field MRI. IEEE Trans Biomed Circuits Syst. 2018.” suggest compensating for the impedance with suitable lumped element terminations at the end of the open stub line. The selection of suitable lumped elements in a multinuclear setting for impedance compensation of the open stub lines is the overarching theme of the manuscript.

We have clarified the sentence by referring to the lumped element terminations for compensation in the sentence. **(R2-16)**

17) Line 75: Please provide related references for the sentence “ To date, switch matrix design has mostly been concerned with routing the signals of the proton Larmor frequency”. Just for curiosity, how many proton switch matrix design implemented on MRI systems?

Switch matrixes are currently only employed in MRI systems with clinical field strength and where coil arrays with a large number of receive elements only exist for the proton frequency. We have extended the description of the current state-of-the-art in switch matrix implementations so as to give the reader more detailed background information **(R2-17a)**. We have also referenced some designs that explicitly state compatibility with X-nuclei operation **(R2-17b)**.

The existing number of matrix implementations is probably similar to the number of MR vendors active on the market as each vendor has a proprietary solution as to how best to route the receive signals. We have tried to add this point in the revised manuscript by referring to the corresponding patent publications.

18) Line 75: What could be the reason(s) of the limited switch matrix design implementation on only proton? Could it be applied in broadband in frequency?

As discussed, the number of X-nuclei receive arrays for clinical field strengths is limited. Thus, the emphasis in switch matrix implementations for clinical field strength is first on protons. However, we agree that most clinical scanners can switch X-nucleus signals since they do not provide dedicated X-nuclei coil connectors. Thus, they need to route a single (or low number of X-signals) to the receivers that are then used to digitize both, proton and X-nucleus signals.

An in-depth discussion would probably need to emphasize that most clinical MRI machines use dedicated coil connectors that bundle a number of receive channels, PIN diode drive signals, digital interface lines and transmit connections. Here, the TX connector is not usually multiplexed – there is a dedicated plug that provides 1H/19F (because the proton amplifier also covers the nearby 19F frequency range) excitation power and one that is used for the other X-nuclei. Usually, the receive signals from the X-nucleus coils are then interfaced to the same connector on the patient table that supplies the TX power. Consequently, the switch matrix could incorporate signal routing for proton and X-nuclei on some (input) channels and for protons only on the other.

We have tried to take the above into account by reformulating the statement in the revised manuscript. **(R2-18)**

19) Line 78: Could the authors correct the sentence “ As multichannel X-nucleus coil

arrays provide the same array SNR benefits...”? Its meaning is not clear.

Generally, using coil arrays provides benefits in terms of SNR (at least close to the location of the single array elements) compared to the use of volume coils. This is due to the fact that the volume coils receive noise from the entire sample inside their sensitive volume, whereas small surface elements only receive noise from a smaller sample region. We apologize for our lack of clarity and have rephrased the statement. **(R2-19)**

20) Line 81: Please correct the placement of “suitable” in the sentence. The meaning of

the sentence is not clear.

Thank you for your suggestion. In the sentence, we have selected the adjective 'suitable' to describe “having sufficient or the required properties for a certain purpose or task; appropriate to a certain occasion”. We have had the sentence rechecked by native speakers and feel reassured of its correctness. **(R2-20)**

21) Line 83: The limitations to design a switch matrix in broadband is not clear so far.

Please include in the introduction section with related references.

The authors have included a statement that a switch matrix is currently not implemented in commercially available ultra-high-field (≥ 7 T) MRI systems **(R2-21a)**. Thus, the necessity for the design task presented in this manuscript is underlined.

As discussed before, X-nucleus imaging/spectroscopy is a major field of application in UHF MRI. This, as well as the ongoing trend for high cannel count X-nucleus arrays, makes the design of a switch matrix capable of operating at multiple frequencies desirable.

We have also added a short example of why a broadband or frequency-independent implementation using GaAs SPDT switches is technologically challenging for matrices displaying maximum flexibility in signal routing and operated at UHF. **(R2-21b)**

22) Line 91: Please provide the elements of the switch matrix topology. Its detailed

description is required if any changes was applied on the hardware design.

As in our previous publication, the switch matrix design is based on PIN diode switches. This time, however, it is designed to operate at the MR frequencies of multiple nuclei. We have added this detail in the revised manuscript. **(R2-22)**

23) Fig1, Page 25: Is there any added design parameters to make the PIN-diode based

switch matrix in broadband?

If the definition of broadband in this question implies operation at a continuous range of frequencies, the only solution would be to configure each PIN diode-based switch as an SPDT switch by using multiple PIN diodes per switch. This, in turn, has a similar effect on insertion loss as the example given in response to your comment #21 **(R2-21b)**. In the worst case, the receive signal has to cross a large number of switches, resulting in significant signal attenuation and degradation of the SNR.

24) Line 149: Could the authors give the optimum “insertion loss”? What was the ideal

insertion loss aimed?

The “optimum” insertion loss would be losses through attenuation on the transmission lines only. In the final large-scale matrix designed in this work, the highest loss due to attenuation along the longest transmission line is 3.28 dB – as stated at the end of the “result” section.

The maximum insertion loss that one is ready to accept in a switch matrix depends on the configuration of the receiver chain of the specific MR system. Generally, the losses of the switch matrix should be low enough to have a negligible influence on the overall noise figure of the receive chain. We have measured the influence of attenuation (at the location of the switch matrix) on image SNR on our 9.4T animal scanner using a linear polarized birdcage resonator. The results are displayed in Figure 3, which shows that an IL as low as possible is generally desirable. **(R2-24)**

The limit can of course also be derived theoretically using the formula below

$F_{tot}=F_{1}+\frac{\left( F_{2}-1 \right)}{G_{1}}+\frac{\left( F_{3}-1 \right)}{\left( G_{1}G_{2} \right)}+ \cdots+ \frac{\left( F_{N}-1 \right)}{\left( G_{1}G_{2}\cdots G_{N-1} \right)}$	(1)

We have not included this information in the manuscript itself, but some common figures are assembled here. In MRI systems, an estimation of the noise contributions using common data – F_coil_ approx. 1.12 for a coil that is 90% sample loss dominated (A. Kumar, W. A. Edelstein, and P. A. Bottomley, "Noise figure limits for circular loop MR coils," Magnetic resonance in medicine, vol. 61, no. 5, pp. 1201-1209, 2009), a preamplifier with a gain of 27 dB and a NF of 0.7 dB, approx. 5 m of cable between coil and switch matrix – the first three stages amount to: F_1_ = F_coil_ = 1.12, G_1_ = 1, F_2_ = F_preamp_ = 10^0.07^ = 1,175, G_2_ = G_preamp_ = 501, F_3_ = F_cable_ = 10^0.25^ = 1.778 and G_3_ = G_cable_ = 0.562.

It should be noted that a full switch matrix of the size discussed in the manuscript gives a worst-case insertion loss of approximately 47 dB. This is assuming that when using an SPDT switch with an IL of 0.25 dB per switch, the overall IL loss of the longest signal path is 0.25 * 127 * 64 – which is sufficient to completely nullify the receive signal according to the exemplary measurements carried out on the animal system.

25) Line 157: What is the motivation of choosing a switch matric with 128x64 channels?

How is this number translated into the “receiver channels” or “receive elements”?

An M x N switch matrix has M inputs and N outputs. We have introduced this notation in the methods section **(R2-25a)**. Consequently, the 128x64 matrix investigated here has 128 inputs and 64 outputs. The dimension chosen here is, to some extent, arbitrary – but it is oriented on the maximum number of receive elements reported in the literature (Schmitt et al.) and a modern MRI system equipped with 64 receiver channels. This number of receivers can be integrated into commercial systems from the major vendors, although the custom integration of more receiver channels has also been reported, e.g. for the 128-channel coil referenced above. However, we feel that the matrix size selected for the large-scale matrix in this manuscript is realistic, and, in response to your comment, we have added a short motivation for the matrix size in the text. (**R2-25b)**

26) Line 237, Discussion section: this section misses the discussion of the methods used in this manuscript in broader MRI context. I strongly recommend the authors to include the contribution of this novelty into the MRI image quality and acquisition time. Even the optimization methods have been explored here, its contribution into the receiver power and receiver elements design were not addressed.

The authors have added a discussion on the effect of inserting the switch matrix and its impact on the SNR of images **(R2-26)**. While the insertion loss has some influence on receive signal amplitude, this effect is less relevant as adjustable gain in the (analogue or digital) receiver chain can compensate for it. Thus, the measurements presented in Figure 3 of the revised manuscript include an overall description of the effect of signal attenuation through the insertion of the matrix. A discussion on the optimisation methods employed here has also been included by referencing multi-objective optimization strategies and their application in other areas of MRI.

Overall, the discussion section has been extended to address the concerns raised in your comment, as well as those from the other reviewers. We now feel that a more complete view has been presented, allowing the reader to assess the results presented in the manuscript.

## Reviewer #3

The authors compare several optimization methods for address the non-convex problem of optimizing RF PIN diode switch performance for MRI receive chains that have multi-nuclear capability. The authors show how adding a small number of discrete impedance elements to the end of stubs in a cross-bar switch network can greatly improve insertion loss by compensating for the variable lengths of stubs, which depend on which output a given input is connected to. The optimization method provides a design for 128 input ports and 64 output ports that works at several X-nuclei frequencies and provides maximum insertion losses that are at most 0.6 dB above the baseline loss for the transmission line in the switch itself. I think the strength of the paper lies in its comparison of several optimization methods that can handle non-convex objective functions, and in the comparatively simple switch network design that requires only four impedance options at the end of each stub to provide adequate degrees of freedom for achieving good impedance match across a variety of stub lengths and operating frequencies.

The authors thank the reviewer for their favourable evaluation of the manuscript.

For general context, I think the authors should add a brief discussion in the introduction about how multi-nuclear signal reception is handled on existing ultra high field scanners that have X-nuclei capability. On these scanners you can choose which nucleus to measure on each receive channel on the patient table socket. Do the authors know what kind of switching hardware is used on these scanners? What about these existing switches is suboptimal? Too much insertion loss for certain configurations? A better discussion of the shortcomings of existing technology would help motivate the switch optimization framework described in the present manuscript.

A number of current commercial UHF systems do not use a switch matrix and hard route the connectors on the patient table to the receivers, e.g. Magentom 9.4T and Magnetom Terra (both: Siemens Healthineers, Erlangen, Germany). This information has been added in the revised manuscript **(R3-1a)**. In this way, losses through the switch matrix are eliminated and X-nucleus coils can connect to any socket on the patient bed, although these are limited in number. However, the combination of, for example, a CP proton coil with a 32-channel sodium array is not feasible when the system is limited to 32 receivers **(R3-1b)**.

Although broadband versions of switch matrixes using SPDT switches exist, one of their major shortcomings is the insertion loss encountered along the longest RF path. On this path, the receive signal has to pass through a large number of RF switches. Although each of the switches does have a very low insertion loss itself, the overall number of switches accumulates the losses to a substantial number. A more precise investigation of this effect has been carried out experimentally and is shown in Figure. 3(compare also **(R2-24**)).

Finally, as far as existing switch matrix implementations are concerned, several patent publications have now been referenced **(R3-1c)**. Since most of the switch matrixes implemented in commercial MRI systems use proprietary implementations, little other information is available. However, in response to **(R2-24),** we have inserted a calculation of IL for a large-scale matrix – which results in a worst-case IL of more than 40 dB when a large number of SPDT switches – each with a small IL itself – has to be crossed by the RF signal.

In previous work the authors show some loss of image SNR due to transmission line losses (Ref. 19). How much does the switch insertion loss impact the final image SNR (and the total system noise figure)? The signals have already passed through a preamplifier, so subsequent losses have less impact on the overall noise figure of the system compared to losses at the coil itself before amplification.

It is certainly true that the low noise amplifier (LNA) inside the receive coils is the dominant component for SNR degradation in the receiver chain. We therefore refer the reviewer to the discussion of R2-24 in this document.

For further elucidation on the subject, we have added measurements results from a 9.4 T animal scanner depicting SNR versus attenuation – Figure 3. As the birdcage coil used for the experiment uses the so-called 'body-coil path' of the system, the preamplifiers are not directly on the coil but are spatially removed to a TR switch unit just outside the magnet. This configuration has been shown to be sensitive to attenuation – even though the attenuator is placed behind the preamplifiers. A second evaluation with a coil with integrated preamplifiers (novel Figure 4) shows a similar picture. With respect to this measurement, keeping the overall IL of the switch matrix as low as possible is a desirable goal. **(R3-2)**

Is there a closed-form expression for the insertion loss as a function of the stub lengths and termination impedances? This would be helpful for readers to see since it will provide more intuition for the optimization problem that is being solved. The authors reference “analytical transmission line formulas” but do not include them in the paper. Equation 1 is not particularly helpful by itself. I would include more detail here so that readers don't have to consult other publications to understand the objective function fully.

There is no closed form solution for the loss of the IL for different stub lengths/terminations. Instead, the IL has to be computed by transforming the termination resistance of the stub lines to the switch matrix junction. The resulting impedance is computed from the three parallel impedances present at this point and the transformation of this to the input of the switch matrix.

As suggested by the reviewer, for the readers' convenience, we have added the formula for the impedance transformation along a transmission line **(R3-3a)**. We have also explained how to derive the transmission line properties from the geometry of the line and material properties of the substrate. However, since the formulas presented by Hammerstad and Jensen require calculation of several intermediate parameters and are quite bulky, we have not reprinted them here. Instead, a comprehensive textbook is referenced that summarizes the formulas. **(R3-3b)**. We have also uploaded the source code of the python script used to optimize the switch matrix into a public repository so that interested readers can refer to this for the in-depth calculations.

Some experimental results would help validate the proposed optimization framework. It would make the paper more convincing if the authors could show that a prototype switch circuit indeed works for all the multinuclear frequencies of interest with the predicted insertion loss in each channel. A small board (such as 5x5 channels) should be sufficient for proof of concept. This would significantly strengthen the manuscript. I don’t think actual NMR measurements are needed, just bench top validation using a network analyzer to verify that the predicted insertion loss at each frequency for each switch configuration matches the experimental results.

For a small-sized matrix (6x3), the compensation elements were determined using the proposed algorithm with an allowance of 1000 iterations. The elements, transmission line dimensions and distances were subsequently put into a circuit simulator (QUCS – available from qucs.sourceforge.net) and the IL loss for the proton frequency was computed in the simulator. The results are displayed in S4 Figure. As can be seen, our formulas slightly underestimate the losses with respect to the losses calculated from QUCS but there is generally good agreement between both. **(R3-4)**

I would try to include more key results in the abstract. For example, what is the average insertion loss for a naive switch with no impedance compensation elements at the end of each stub, as compared to the optimized result that shows no more than 0.6 dB of added insertion loss above the basic transmission line losses.

We have included a statistical evaluation of the insertion losses which includes mean insertion loss and standard deviation of the insertion loss. **(R3-5)**

3.28 dB of transmission line loss for the proton frequency seems quite high. Is a low-loss dielectric substrate used?

The calculations have been carried out with a loss tangent of 0.022, which is the loss tangent for standard FR4. Certainly, this could be improved by selecting a lower loss substrate. However, as this work was only a proof-of-concept for the switch matrix design, we have chosen the low-cost implementation using a FR4 substrate.

How will the switching between the different stub termination impedances be implemented in practice?

In a product implementation, SPST PIN diode switches can equally be employed to connect the designated termination impedance to the stub lines. This information has been added in the revised manuscript. **(R3-7)**

“In this context, designing a switch matrix suitable for operation with protons and the most common X-nuclei at high-field is desirable.” Can the authors clarify exactly what role the switch will play on a multi-nuclear scanner? For example using the same plug on the patient table to route different coil channels to different X-nucleus receiver channels inside the scanner.

We have added an example of where a switch matrix capable of operating at multiple frequencies would be desirable in the revised manuscript. Another example that involves a more sophisticated RF coil array, would be to connect the output of a high-channel-count, double-tuned receive array to the available MR receivers alternately. An example of such a coil has just been published by Rowland et al. Whole brain 31 P MRSI at 7T with a dual-tuned receive array. MRM 2020. This reference has also been added in the revised manuscript. **(R3-8)**

Can the authors discuss why the problem is non-convex? What properties of the objective function make it non-convex?

Many thanks for raising this point and we apologize for omitting this explanation. At least two possible solutions exist to minimize the IL since the IL for each junction in the switch matrix is a magnitude function and depends on the two complex terminations of the associated transmission lines. Thus, at least two minima exist in the optimization landscape for each junction, which makes the optimization problem become non-convex. **(R3-9)**

I think it would be good to include more details about the circuit design from Ref. 19 for readers who don’t have time to consult that reference. For example, I found it helpful to look at Fig. 2c from Ref. 19. At a minimum, I would draw the PIN diode circuit symbol in at least one of the figures to show how it functions in a crossbar switch, to help readers appreciate why the stubs remain after an input is connected to a desired output channel.

We thank the reviewer for pointing this out. We have included the general topology of the crossbar switch matrix in the modified Figure 1 for a convenient reference. **(R3-10)**

Please expand caption for Figure 2 and add appropriate labels to figure. Explain explicitly what is meant by “Input 1, 2, 3…”, “Channel 1, 2, 3, …” and the letters “A, B, C, …”

We have added the definition for input and channel in the figure caption. The letters denote transmission line stubs – which has also been added to the figure caption. **(R3-11)**

Figure 1. Is “voltage controlled RF switch” the same as PIN diode switch? A PIN diode is really “current-controlled” since it requires current to be forward biased, so it might be more accurate to call it current-controlled or just a “PIN diode-actuated switch”.

Our apologies – but we can’t really find any reference to “voltage controlled” in the manuscript.

On the subject, we agree that PIN diodes are current-controlled in forward bias, however, reverse bias requires the application of a negative voltage across the PIN diode junction. Thus the suggest formulation suggested by the reviewer is the most appropriate one.

Figure 2 caption. Please add more detail to the caption (and manuscript text), for example describing what is denoted with the red text and red outline. Also why aren’t the other switch cases considered, for example Input 1 going to Channels 1-4? Shouldn't all inputs be able to connect to all output channels? This is not clearly explained in the text. Are the values reported in Fig. 2 calculated or measured experimentally?

The authors have added the requested information to the figure caption **(R3-13a)** and also in the text **(R3-13b)**. All values presented in Figure 2 are measured insertion losses on a small prototype PCB which used fixed connections between inputs and outputs for the sake of reducing the experimental implementation burden. Care was taken to ensure that the fixed routing represented connections with different stub line lengths. Interestingly, the highest insertion loss is not always encountered in the connection with the longest open stub lines. This, of course, depends on the actual implementation (physical dimensions) of the switch matrix and the operating frequency could be different for other implementations.

I would recommend adding more labels and details to Figures 3-5. Please add labels for the rows and columns of each matrix. And please add more detail to the caption explaining what is going on in the matrices/tables. I assume the two entries in each cell correspond to the selected impedance for the two stubs realized on the two sides of the PCB for minimizing the insertion loss. Same for Figure 6, please add labels to each panel in the figure. I assume the dimension of the arrays is 128x64? Please clarify that the values are simulated.

We have removed Figures 3-5 and have placed these in the supporting material. The corresponding figure captions have been adapted to include more detail as suggested by the reviewer. The same holds for the caption of Figure 6 (now Figure 5). **(R3-14)**

Is the 17O insertion loss generally lower than the other isotopes because of its lower Larmor frequency?

As far as Figure 2 is concerned, the switch matrix is simply too small to display a substantial transmission line length at the ^17^O frequency. The original Figures 3 to 5 show that for the larger sized 16 x 8 matrices the transmission line stubs are also usually left unterminated at the low resonance frequency of ^17^O. Once the matrix size becomes larger, terminations are required and are computed by the proposed design algorithm.

Although we agree that the ^17^O measurements are the least affected, we feel that the inclusion of this data is beneficial for illustrative purposes.

“…allowed was limited from 300 nH inductive to 200 pF capacitive, which covers available lumped, high frequency elements with suitable self-resonances and quality factors.” I assume these parts are non-magnetic and that this limits the readily available range of component values. Or were there other factors motivating the specific choice of impedance matching component values?

Thank you for raising this point. Actually, the available range of non-magnetic components is slightly larger. We commonly employ capacitors from ATC, series B, in our lab. Although the range extends to 1000 pF, the RF performance of the larger values drops quickly in available Q, especially for higher frequencies. For example, the Q curve in the datasheet at 500 MHz ends at around 400 pF with a Q of 20. From experience, we usually limit the range in MR coils to around 100 pF for the proton frequency at 9.4 T.

Similarly, most of the inductors we use in MR coils employ hand-wound air-core implementations. Here, Q is even more of an issue. A good example of the performance of inductors is to look at the “air-core” series from Coilcraft. The highest value available is 538 nH – but this value is not suitable for operating frequencies like the 9.4 T proton resonance frequency. Consequently, 300 nH, as assumed here, already represents a high value.

Finally, it should be noted that the values chosen by the proposed design algorithm are usually below theses maximum limits – which can be seen from the original Figures 3-5. Thus, we feel that setting these boundaries imposes a valuable compromise between practical implementation and the need to avoid limiting the search space too much.

p. 7: “128x64 channels…” What does each dimension refer to? 128 inputs and 64 outputs?

We have now defined this notation in the “Methods” section **(R3-16)**. Thus, as correctly hinted by the reviewer, this is a switch matrix with 128 input channels and 64 outputs.

Abstract

“…high field systems…”

Thank you for careful proofreading. We have corrected this typo. **(R3-17)**

“As multichannel X-nucleus coil arrays provide the same array SNR benefits as in proton imaging, there is compelling motivation to also use coil arrays in these SNR-starved applications.”

Thank you for suggesting this elegant formulation which we have copied into the revised manuscript. **(R3-18)**

p. 4: “However, with the increased availability of ultra high field scanners, the benefits of X-nuclei (non-proton, resonating at different frequencies) imaging is likely to attract increased interest.”

One more, we appreciate the skilled formulation suggested by the reviewer. (R3-19)

p. 5: “…as previously described in Ref. (19) for the connections…”

We have now added “previously” in the revised manuscript. **(R3-20)**

p. 7: “tuple” is somewhat uncommon usage here.

We agree and have replaced “tuple” with “number”. **(R3-21)**

p. 9: “…while other algorithms converged to significantly worse solutions…”

We thank the reviewer for careful proofreading and have corrected the typo. **(R3-22)**

p. 9: “This task was based on a…”

See above. Corrected. **(R3-23)**

p. 12: “…lends reasonable credibility only to the results obtained…”

The sentence was modified as suggested. (R3-24)

# PLOS EDITORIAL COMMENTS

1. Please ensure that your manuscript meets PLOS ONE's style requirements, including those for file naming. The PLOS ONE style templates can be found at http://www.plosone.org/attachments/PLOSOne_formatting_sample_main_body.pdf and http://www.plosone.org/attachments/PLOSOne_formatting_sample_title_authors_affiliations.pdf

2. We note that Figure 1 in your submission contain copyrighted images. All PLOS content is published under the Creative Commons Attribution License (CC BY 4.0), which means that the manuscript, images, and Supporting Information files will be freely available online, and any third party is permitted to access, download, copy, distribute, and use these materials in any way, even commercially, with proper attribution. For more information, see our copyright guidelines: http://journals.plos.org/plosone/s/licenses-and-copyright.

The authors have redrawn Figure 1 for improved clarity as well as to avoid potential copyright conflicts.

3. Thank you for stating the following in the Competing Interests section:

"I have read the journal's policy and the authors of this manuscript have the following competing interests: NJS and JF are co-founders of Affinity Imaging GmbH a spin-off company that manufactures high field MRI coils for research purposes."

Please confirm that this does not alter your adherence to all PLOS ONE policies on sharing data and materials, by including the following statement: "This does not alter our adherence to PLOS ONE policies on sharing data and materials.” (as detailed online in our guide for authors http://journals.plos.org/plosone/s/competing-interests). If there are restrictions on sharing of data and/or materials, please state these. Please note that we cannot proceed with consideration of your article until this information has been declared.

Please include your updated Competing Interests statement in your cover letter; we will change the online submission form on your behalf.

Please know it is PLOS ONE policy for corresponding authors to declare, on behalf of all authors, all potential competing interests for the purposes of transparency. PLOS defines a competing interest as anything that interferes with, or could reasonably be perceived as interfering with, the full and objective presentation, peer review, editorial decision-making, or publication of research or non-research articles submitted to one of the journals. Competing interests can be financial or non-financial, professional, or personal. Competing interests can arise in relationship to an organization or another person. Please follow this link to our website for more details on competing interests: http://journals.plos.org/plosone/s/competing-interests

The authors have updated the declaration of competing interests in the cover letter as suggested.
