# Supplementary material for: Optimization of high-channel count, switch matrices for multinuclear, high-field MRI
Source: PLoS One. 2020 Aug 17;15(8):e0237494. doi: 10.1371/journal.pone.0237494 (PMC7430713; doi:10.1371/journal.pone.0237494)
Supplement: Attachment — Submitted filename: Respose_To_Reviewers_Rev2.docx [file pone.0237494.s007.docx]

# Review Comments to the Author

## Reviewer #1:

I think the manuscript has been significantly improved. The information provided, with the evaluation of loss in SNR with different nuclei with different attenuation, puts into context the improvements in IL with the switch matrices. The additional literature references on the algorithms and availability of the used algorithm provide a way to evaluate the success of the implementation here as well. The comments listed below can be corrected in editing and don’t require another review.

We thank the reviewer for his favourable evaluation and are satisfied that the text could be improved with the help of the suggestions from the first revision.

Minor Comments:

In abstract, instead of writing “For a matrix with 128 inputs and 64 outputs a realization is proposed that displays a worst-case insertion loss of just 0.6 dB above that of the attenuation of the longest transmission line.” Write “For a matrix with 128 inputs and 64 outputs a realization is proposed that displays a worst-case insertion loss of {the insertion loss}”

We have changed the sentence as suggested (R1-1).

page 3, row 92: have defined “single-pole doublethrow (SPDT) twice”

Searching through the manuscript we found only one definition of SPDT and one of SPST. If we missed something during this revision, we are happy to modify this during final editing.

The introduction has been improved greatly and reads very well, with a more than extensive review of the literature.

The authors are pleased that the introduction now reads smoothly.

Page 11, row 256: units for surface roughness are missing, also which is being quoted, peak-to-valley roughness (Rz), average roughness (Ra), or RMS roughness (Rrms)?

The value is RMS roughness. We have added this information in the text for clarity (R1-4).

## Reviewer #2:

Thanks for addressing all questions and comments.

We thank the reviewer for once again carefully reading the manuscript.

## Reviewer #3:

Supporting Figure 4 has no labels accompanying the table/matrix. A label or title would be helpful.

We apologize for not having added captions to the plot. We have added captions to the revised plot.

For a PIN diode switch to be effective, a good bias tee must be used to inject the bias current into the PIN diode path, while blocking RF from the DC bias path. What kind of choke is used that is effective for blocking all the X-nuclei frequencies? Typically, an inductor with appropriate self-resonance frequency a little bit higher than the Larmor frequency is used, but for the case of a switch handling multiple Larmor frequencies, this is more complicated. An inductor with suitably high inductance might work decently for all the frequencies used, but this might require a ferrite component. Some basic design details might be helpful to other investigators wishing to reproduce the work (using non-magnetic circuit components).

Up to now, we have employed IM4 type inductors from Vishay. The non-magnetic version (no ferrite core) of this series has a maximum value of 4.7 μH with a self-resonance that is just above 400 MHz so that we also used it in some 9.4 T proton coils. One should take care to shorten the leads as much as possible, however, since otherwise the self-resonance frequency decreases. On the other hand, this value also presents a suitably large impedance for lower Larmor frequencies.

One should also take note that we are currently seeking alternative inductors as the product has been declared obsolete by the manufacturer.

In the current version of the manuscript we have refrained from adding circuit implementation details such as component selection or bias driver design. Although these might be of interest for individual readers the amount of information required for the actual implementation is beyond the scope of the manuscript. This is why we focus on the optimization of the geometrical layout of the switch matrix. In addition, a good number of publications exist that discuss the implementation details of PIN diode switches. One of my favourite remains the “THE PIN DIODE CIRCUIT DESIGNERS’ HANDBOOK” available at <https://www.ieee.li/pdf/essay/pin_diode_handbook.pdf>.

The authors write on p. 6, line 122, that on some systems, including 7T systems, "the switch matrix was removed and the signal was routed directly without the possibility to switch it to a different receiver unit". I'm still confused about this. On the Siemens Magnetom 7T at my institution, the coil file specifies whether the pins on Plug 3 should be used for, say, 1H or 31P signal reception. There is presumably a switch somewhere in the receive chain to route the signals accordingly.

During the first revision –where the point of fixed connections in the UHF switch matrix of Siemens scanners has also been raised – we reconfirmed this statement in the manuscript by directly inquiring with the Siemens’ UHF team. The unit called RCCS in Siemens scanners, which is an abbreviation of “receive coil channel selector” does indeed not contain a switch matrix for their 7 T and 9.4 T scanners. Instead plugs #1 and #2 are hard routed to receiver cassette #1 with 16 receive channels and plugs #3 and #4 are directly connected with receiver cassette number #2. Thus, no channel selection is possible in the sense known from clinical scanners.

However, as you describe, frequency switching of the receiver channels is possible to support multinuclear operation. Thus, receiver cassette #2 (connected to plus #3 and #4) can be switched in frequency to detect 31P signals, for example.
